# Identification and characterization of Coronaviridae genomes from Vietnamese bats and rats based on conserved protein domains

**DOI:** 10.1093/ve/vey035

**Published:** 2018-12-15

**Authors:** My V T Phan, Tue Ngo Tri, Pham Hong Anh, Stephen Baker, Paul Kellam, Matthew Cotten

**Affiliations:** 1 Virus Genomics, Wellcome Trust Sanger Institute, Hinxton, Cambridge, UK; 2 Department of Viroscience, Erasmus Medical Center, Rotterdam, The Netherlands; 3 Wellcome Trust Major Overseas Programme, Oxford University Clinical Research Unit, Ho Chi Minh City, Vietnam; 4 Department of Infection and Immunity, Imperial College London, London, UK; 5 Kymab Ltd, Babraham Research Campus, Cambridge, UK

**Keywords:** virus classification, machine learning, random forest, protein domains, Pfam, profile Hidden Markov model

## Abstract

The *Coronaviridae* family of viruses encompasses a group of pathogens with a zoonotic potential as observed from previous outbreaks of the severe acute respiratory syndrome coronavirus and Middle East respiratory syndrome coronavirus. Accordingly, it seems important to identify and document the coronaviruses in animal reservoirs, many of which are uncharacterized and potentially missed by more standard diagnostic assays. A combination of sensitive deep sequencing technology and computational algorithms is essential for virus surveillance, especially for characterizing novel- or distantly related virus strains. Here, we explore the use of profile Hidden Markov Model-defined Pfam protein domains (Pfam domains) encoded by new sequences as a *Coronaviridae* sequence classification tool. The encoded domains are used first in a triage to identify potential *Coronaviridae* sequences and then processed using a Random Forest method to classify the sequences to the *Coronaviridae* genus level. The application of this algorithm on *Coronaviridae* genomes assembled from agnostic deep sequencing data from surveillance of bats and rats in Dong Thap province (Vietnam) identified thirty-four *Alphacoronavirus* and eleven *Betacoronavirus* genomes. This collection of bat and rat coronaviruses genomes provided essential information on the local diversity of coronaviruses and substantially expanded the number of coronavirus full genomes available from bat and rats and may facilitate further molecular studies on this group of viruses.

## 1. Introduction

The *Coronaviridae* family comprises enveloped positive-sense single-stranded RNA viruses of the order *Nidovirales* with a genome of up to 32 kb in length. The family is divided into *Coronavirinae* and *Torovirinae* sub-families, which are further divided into six genera: *Alphacoronavirus, Betacoronavirus, Gammacoronavirus, Deltacoronavirus, Torovirus*,* and Bafinivirus.* While viruses in the genera *Alphacoronaviruses* and *Betacoronaviruses* infect mostly mammals, the *Gammacoronavirus* infect avian species and members of the *Deltacoronavirus* genus have been found in both mammalian and avian hosts (de Groot 2012; Drexler, Corman, and Drosten 2014).

Coronaviruses (CoVs) cause a range of respiratory, enteric, and neurological diseases in human and animals. In human CoV infections, the severe acute respiratory syndrome coronavirus (SARS-CoV) and Middle East respiratory syndrome coronavirus (MERS-CoV) cause severe respiratory tract disease with high mortality rates, and there is strong evidence of zoonosis for both viruses (Hu et al. 2015; Dudas et al. 2018; Leopardi et al. 2018). Given such zoonotic movement, detailed descriptions of the *Coronaviridae* in broad animal reservoirs that may cross the host barriers to cause diseases in humans are important; many of these Coronaviridae strains in animal reservoirs could represent uncharacterized strains and be missed by conventional diagnostic assays.

Advances in nucleic acid sequencing technology (commonly termed Next-Generation Sequencing, NGS) are providing large sets of sequence data obtained from a variety of biological samples and allowing the characterization of both known and novel virus strains. Algorithms that can accurately and rapidly detect and classify low-frequency virus sequences amidst a high-sequence background are useful. The desired features of these classification algorithms are the ability to rapidly process large number of sequences and to accurately identify more distantly related sequences. Use of such tools in the field during outbreak sequencing is common, thus methods that are stand-alone requiring no internet connection are desirable.

All viruses encode a collection of proteins required to ensure self-replication and persistence of the encoding virus. Enzymes for genome mRNA production and genome replication, proteases for protein maturation, proteins for genome encapsidation, and proteins for undermining the host antiviral responses can all be identified conserved protein motifs or domains. Likely because of selective pressures, viral genomes are streamlined and the functional protein content encoded by viruses is much higher than for a cellular organisms. Thus, describing a viral genome by the collection of encoded protein domains is a potentially useful classification method that we would like to explore in more detail.

Profile Hidden Markov Models (HMMs) provide a probabilistic framework for describing multiple sequence alignments that can reveal position-specific patterns (Krogh, Mian, and Haussler 1994; Eddy 1996, 1998; Durbin et al. 1998; Sonnhammer et al. 1998). The Pfam protein families database (Finn et al. 2016) of >16,000 protein domains is available (Pfam 31.0 at http://Pfam.xfam.org/). Within the Pfam collection, each domain family is defined by a manually selected and aligned set of protein sequences, which is used to construct a profile HMM of the domain. The HMM domain concept and search algorithms for generating and detecting profile HMMs have gone through a number of refinements and a current rapid implementation for finding profile HMMs in novel sequences is HMMER3 (http://hmmer.org/, Eddy 2011).

A number of strategies have been developed to use protein domains for virus sequence classification. The Virus Pathogen Resource (ViPR) site has a useful compilation of Pfam domains found in specific virus families (https://www.viprbrc.org/brc/home.spg? decorator=vipr) however the catalog is currently limited to fourteen virus families while the International Committee on Taxonomy of Viruses (ICTV) currently recognizes ninty-six virus families (Lefkowitz et al. 2018). The use of profile Hidden Markov Models (HMMs) for virus classification and discovery was recently reviewed (Reyes et al. 2017). The use of an HMM structure as the basis for sequence classification has the potential to identify more distant members of protein domain family. Both Metavir (Roux et al. 2011) and VirSorter (Roux et al. 2015) make extensive use of protein domains as part of effective virus classification algorithms. The implementation VirSorter is primarily focused on identifying novel bacteriophage sequences. MetLab (Norling et al. 2016) and vFAM (Skewes-Cox et al. 2014) methods have demonstrated the utility of such a protein domain classification approach. ClassyFlu (Van der Auwera et al. 2014) builds influenza subtype specific profile HMM-defined protein domains for the HA coding region and then uses this database of HMMs to classify test influenza HA segments.

We describe here a strategy using Pfam protein domains as the basis for identifying and classifying *Coronaviridae* genomic sequences. We show that the method can be used for rapid identification of *Coronaviridae* sequences in *de novo* assembled contigs, although sensitivity requires longer, ideally genome-length contigs. If sufficient sequence across the virus genome is available, the method can provide virus classification to the genus level. We then employ this method to identify fourty-five novel *Coronaviridae* genome sequences from random-primed deep sequencing data from bats and rats sampled from Dong Thap province of Vietnam.

## 2. Materials and methods

### 2.1 Study setting and design

**Figure 1.**
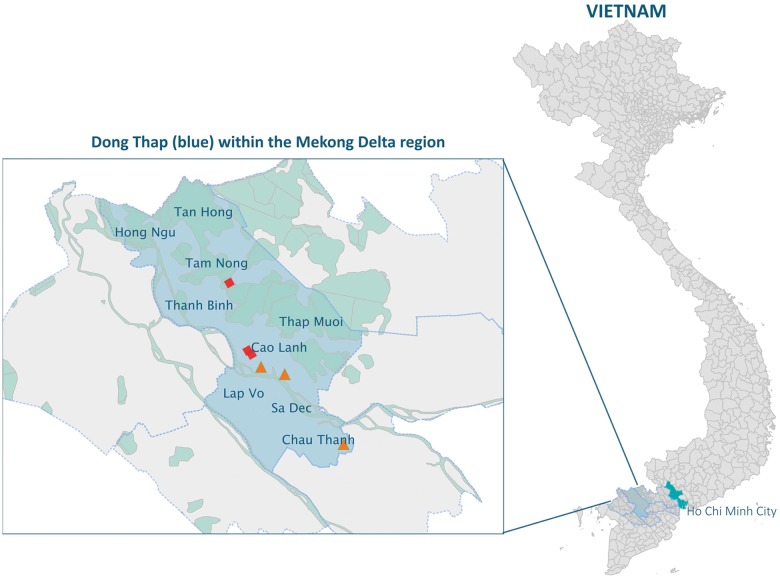
Location of the sampling sites. The right panel shows the map of Vietnam, the left inset shows the Dong Thap province (marked in blue and separated by dotted lines with neighboring provinces within the Mekong Delta region of southern Vietnam). The Mekong Delta river branches and flooding areas are marked in green. Names of communal regions within Dong Thap province are indicated. Locations of the guano farms where bats were samples are marked with red diamonds, and the locations of rat sampling sites are marked in orange triangles.

Fecal pellets from *Scotophilus kuhlii* bats were collected from roosts on bat guano farms in the Dong Thap province in southern Vietnam, ∼150 km south west of Ho Chi Minh City as shown in the map (Fig. 1). Rat fecal pellets from *Rattus argentiventer* were collected from trapped rice-field rats or from rats purchased in wet markets in Dong Thap (locations are indicated in the map, Fig. 4A). Samples were stored at −80°C until processed for NGS. Approvals for the study were obtained from the Oxford Tropical Research Ethics Committee (Approval No. 15–12) (Oxford, UK), the institutional ethical review board of Dong Thap Provincial Hospital and the Sub-Department of Animal Health, Dong Thap province (Dong Thap, Vietnam).

### 2.2 Sample processing, library preparation, and NGS

Total nucleic acid was extracted as previously described (de Vries et al. 2012; Cotten et al. 2014). In brief, a volume of 110 µl of each sample was centrifuged for 10 min at 10,000 × g. Unprotected (non-encapsidated) DNA in the samples was degraded by addition of 20 U TURBO DNase (Ambion). Remaining nucleic acid was subsequently extracted using the Boom method (Boom et al. 1990). Reverse transcription was performed using non-ribosomal random hexamers (Endoh et al. 2005), and second strand DNA synthesis was performed using 5 U of Klenow fragment (New England Biolabs) followed by phenol/chloroform extraction and ethanol precipitation. Illumina libraries were prepared for each sample, the material was sheared to 400–500 bp in length, separately indexed, and multiplexed at ninety-six samples per HiSeq 2500 run, generating two to three million 250-nt paired-end reads per sample.

### 2.3 *De novo* assembly and identification of *Coronaviridae* contigs

Raw sequencing reads were trimmed to remove residual sequencing adapters and trimmed from the 3′ end to a median Phred score > 35 using QUASR (Watson et al. 2013). The quality controlled reads were assembled into contigs using *de novo* assembly with SPAdes 3.10 (Bankevich et al. 2012). Coverage was estimated for contigs followed by additional filtering for minimum contig size cutoff (300 nt). Final details on the genomes including GenBank accession numbers, sample locations, and collection dates can be found in Table 1. For most of the samples, complete or nearly complete genomes were obtained from the original SPAdes assembly. However, in a subset of samples (usually those with mixed infections or with too high-sequence coverage), SPAdes yielded two or more subgenomic contigs that were manually checked by consulting short reads and re-assembled.

**Table 1.** Genome metrics. Compilation of metrics for the new *Coronaviridae* sequences reported here. The table includes for each genome the genome id and GenBank accession number, and the *Coronaviridae* genus as identified by the tool described in this article. Also included are the host species, the sample collection date, and location data. Finally, the number of short reads in the sample mapping to the complete genome using bowtie2–2.2.3 (Langmead et al. 2009) and the ‘–very-sensitive-local’ settings and the final genome length are reported.

Genome_ID	GenBank^a^	Corona_ genus	Host	Host_species	Farm_ID	Collection_ date	District	Total_qc_ reads(for)^b^	Mapped_ reads^c^	Genome_ length (nt)
16715_23	MH687934	*Alpha*	Bat	*Scotophilus kuhlii*	55	11-Jun-2014	Chau Thanh	2,336,433	57,097	28,864
16715_24	MH687935	*Alpha*	Bat	*Scotophilus kuhlii*	98	17-Jun-2014	Cao Lanh	2,957,491	55,684	29,152
16715_31	MH687936	*Alpha*	Bat	*Scotophilus kuhlii*	98	17-Jun-2014	Cao Lanh	2,419,725	18,696	28,828
16715_32	MH687937	*Alpha*	Bat	*Scotophilus kuhlii*	98	17-Jun-2014	Cao Lanh	2,830,496	60,643	28,297
16715_39_c1	MH687938	*Alpha*	Bat	*Scotophilus kuhlii*	98	17-Jun-2014	Cao Lanh	2,235,269	51,709	28,238
16715_39_c2	MH687939	*Alpha*	Bat	*Scotophilus kuhlii*	98	17-Jun-2014	Cao Lanh	2,235,269	11,042	28,307
16715_45	MH687940	*Alpha*	Bat	*Scotophilus kuhlii*	99	10-Jun-2014	Cao Lanh	2,544,491	12,463	28,170
16715_47_c1	MH687941	*Alpha*	Bat	*Scotophilus kuhlii*	98	17-Jun-2014	Cao Lanh	2,723,720	17,632	28,257
16715_47_c2	MH687942	*Alpha*	Bat	*Scotophilus kuhlii*	98	17-Jun-2014	Cao Lanh	2,723,720	16,651	28,321
16715_5	MH687943	*Alpha*	Bat	*Scotophilus kuhlii*	99	10-Jun-2014	Cao Lanh	2,556,708	32,056	28,272
16715_53	MH687944	*Alpha*	Bat	*Scotophilus kuhlii*	99	10-Jun-2014	Cao Lanh	3,260,590	6,251	28,400
16715_56	MH687945	*Alpha*	Bat	*Scotophilus kuhlii*	99	16-Sep-2014	Cao Lanh	2,596,084	32,895	28,481
16715_61	MH687946	*Alpha*	Bat	*Scotophilus kuhlii*	99	10-Jun-2014	Cao Lanh	2,910,089	3,464	28,173
16715_63	MH687947	*Alpha*	Bat	*Scotophilus kuhlii*	98	17-Jun-2014	Cao Lanh	2,619,814	124,453	29,462
16715_7	MH687948	*Alpha*	Bat	*Scotophilus kuhlii*	55	11-Jun-2014	Chau Thanh	2,304,297	59,108	28,340
16715_76	MH687949	*Alpha*	Bat	*Scotophilus kuhlii*	99	10-Jun-2014	Cao Lanh	2,618,662	36,601	28,232
16715_77	MH687950	*Alpha*	Bat	*Scotophilus kuhlii*	99	10-Jun-2014	Cao Lanh	2,703,803	7,848	28,303
16715_78	MH687951	*Alpha*	Bat	*Scotophilus kuhlii*	55	11-Jun-2014	Chau Thanh	2,540,982	81,202	29,118
16715_84	MH687952	*Alpha*	Bat	*Scotophilus kuhlii*	99	10-Jun-2014	Cao Lanh	2,334,464	5,629	27,225
16715_86	MH687953	*Alpha*	Bat	*Scotophilus kuhlii*	55	11-Jun-2014	Chau Thanh	2,643,553	79,660	28,747
16845_24	MH687954	*Alpha*	Bat	*Scotophilus kuhlii*	55	18-Sep-2014	Chau Thanh	2,794,660	4,334	28,333
16845_47	MH687955	*Alpha*	Bat	*Scotophilus kuhlii*	98	17-Sep-2014	Cao Lanh	2,250,435	5,380	28,437
16845_53	MH687956	*Alpha*	Bat	*Scotophilus kuhlii*	99	16-Sep-2014	Cao Lanh	2,589,540	183,763	28,562
16845_64	MH687957	*Alpha*	Bat	*Scotophilus kuhlii*	55	18-Sep-2014	Chau Thanh	3,114,858	4,264	28,173
16845_87	MH687958	*Alpha*	Bat	*Scotophilus kuhlii*	55	18-Sep-2014	Chau Thanh	2,453,660	6,514	28,054
17819_17	MH687959	*Alpha*	Bat	*Scotophilus kuhlii*	98	12-Nov-2014	Cao Lanh	2,555,417	27,177	28,706
17819_22	MH687960	*Alpha*	Bat	*Scotophilus kuhlii*	55	13-Nov-2014	Chau Thanh	2,546,117	2,306	27,491
17819_4	MH687961	*Alpha*	Bat	*Scotophilus kuhlii*	55	13-Nov-2014	Chau Thanh	2,740,662	5,386	28,380
17819_50	MH687962	*Alpha*	Bat	*Scotophilus kuhlii*	55	13-Nov-2014	Chau Thanh	2,392,592	3,800	28,053
20724_95	MH687963	*Alpha*	Bat	*Scotophilus kuhlii*	99	6-Feb-2015	Cao Lanh	7,568,967	11,331	28,169
20745_10	MH687964	*Alpha*	Bat	*Scotophilus kuhlii*	99	6-Feb-2015	Cao Lanh	1,043,879	6,913	28,210
20745_17	MH687965	*Alpha*	Bat	*Scotophilus kuhlii*	55	13-Feb-2015	Chau Thanh	1,862,525	36,558	28,199
20745_6	MH687966	*Alpha*	Bat	*Scotophilus kuhlii*	99	6-Feb-2015	Cao Lanh	9,273,648	36,639	28,628
20745_8	MH687967	*Alpha*	Bat	*Scotophilus kuhlii*	99	6-Feb-2015	Cao Lanh	12,697,144	4,021	28,038
16715_52	MH687968	*Beta*	Rat	*Rattus argentiventer*	63	14-Nov-2014	Cao Lanh	2,519,218	30,551	31,047
20724_33	MH687969	*Beta*	Rat	*Rattus argentiventer*	65	12-Nov-2014	Tam Nong	536,662	9,918	31,976
20724_34_c12	MH687970	*Beta*	Rat	*Rattus argentiventer*	65	12-Nov-2014	Tam Nong	740,717	8,579	31,038
20724_34_c13	MH687971	*Beta*	Rat	*Rattus argentiventer*	65	12-Nov-2014	Tam Nong	740,717	18,463	31,171
20724_38	MH687972	*Beta*	Rat	*Rattus argentiventer*	65	12-Nov-2014	Tam Nong	8,568,196	14,281	31,334
20724_39	MH687973	*Beta*	Rat	*Rattus argentiventer*	65	12-Nov-2014	Tam Nong	6,703,538	68,652	31,389
20724_43	MH687974	*Beta*	Rat	*Rattus argentiventer*	65	12-Nov-2014	Tam Nong	255,794	27,831	31,224
22054_56	MH687975	*Beta*	Rat	*not available*	62	9-Dec-2013	Cao Lanh	442,567	2,982	29,727
22084_1	MH687976	*Beta*	Rat	*Rattus argentiventer*	65	4-Feb-2015	Tam Nong	29,862,809	100,936	31,068
22084_10	MH687977	*Beta*	Rat	*Rattus argentiventer*	65	4-Feb-2015	Tam Nong	343,514	21,210	31,355
22084_6	MH687978	*Beta*	Rat	*Rattus argentiventer*	65	4-Feb-2015	Tam Nong	28,864,231	121,999	31,289

^a^GenBank accession number.

^b^Total paired eads (after quality control).

^c^Total read mapped to final genome.

### 2.4 Phylogenetic analyses

Global coronavirus reference sequences sharing ≥80 per cent nt similarity to the reported CoVs in this study were retrieved from GenBank in addition to selected reference CoV sequences for comparison. The coding regions of spike protein from all reference and the assembled *Coronaviridae* sequences were extracted and aligned in MUSCLE (Edgar 2004), followed by manual check in AliView (Larsson 2014). The best-fitted nucleotide substitution models were determined in IQ-TREE v1.5.2 using the Akaike Information Criterion (Nguyen et al. 2015). Maximum likelihood (ML) phylogenetic trees were inferred in IQ-TREE employing GTR + I + Γ4 model of substitution, bootstrapping for 1,000 pseudoreplicates. Bootstrap values of ≥70 per cent were considered as statistically significant, and resulting trees were visualized and edited in FigTree v1.4.3 (Rambaut 2016).

### 2.5 Protein domain database

Using the HMMER3 hmmsearch function (Eddy 2011) and the Pfam collection of profile HMM protein domains, we examined all available *Coronaviridae* full genome sequences from GenBank (as of January 2018; *N* = 2,255). A set of seventy-nine Pfam domains were found at least once in a *Coronaviridae* genome and these domains formed the basis for the classification methods explored here. The seventy-nine Pfam domains used for *Coronaviridae* classification and their frequencies in the set of 2,255 *Coronaviridae* genomes are listed in Table 2.

**Table 2.** Pfam domains used for Coronaviridae classification. A compilation of the Pfam domains used for classification of the *Coronaviridae* sequences. All available full *Coronaviridae* genome sequences in GenBank were retrieved using the query ‘txid11118[Organism] AND 25600[SLEN]:48000[SLEN] NOT patent’ to yield a set of 2,255 sequences (3 January 2018). All open reading frames encoding peptides > 100 amino acids in length were were analyzed with the hmmer-3.1b2-hmmscan program (Eddy 2011) and the complete Pfam A database (http://Pfam.xfam.org/, Finn et al. 2016). Domains with E-value < 0.01 were counted and the domain frequencies in the set of 2,255 *Coronaviridae* genomes were reported.

Pfam_id^a^	Name	Frequency^b^	Category^c^
pfam13086	AAA_11	3,712	CATD
pfam06460	NSP13	2,492	CATD
pfam13604	AAA_30	2,438	CTD
pfam01443	Viral_helicase1	2,427	CTD
pfam01661	Macro	2,390	CTD
pfam01600	Corona_S1	6,838	abundant
pfam01601	Corona_S2	5,446	abundant
pfam00937	Corona_nucleoca	4,993	abundant
pfam01635	Corona_M	3,794	abundant
pfam08715	Viral_protease	3,290	abundant
pfam06478	Corona_RPol_N	2,712	abundant
pfam09408	Spike_rec_bind	2,599	abundant
pfam06471	NSP11	2,466	abundant
pfam13087	AAA_12	2,434	abundant
pfam13538	UvrD_C_2	2,432	abundant
pfam05409	Peptidase_C30	2,406	abundant
pfam08717	nsp8	2,398	abundant
pfam08716	nsp7	2,396	abundant
pfam16348	Corona_NSP4_C	2,386	abundant
pfam09401	NSP10	2,384	abundant
pfam08710	nsp9	2,382	abundant
pfam13245	AAA_19	2,067	abundant
pfam00680	RdRP_1	1,868	abundant
pfam03053	Corona_NS3b	1,773	abundant
pfam16451	Spike_NTD	1,461	abundant
pfam16251	NAR	1,142	abundant
pfam11633	SUD-M	855	moderate
pfam03187	Corona_I	586	moderate
pfam03996	Hema_esterase	563	moderate
pfam02710	Hema_HEFG	554	moderate
pfam03262	Corona_6B_7B	534	moderate
pfam02723	NS3_envE	495	moderate
pfam03620	IBV_3C	474	moderate
pfam08779	SARS_X4	414	moderate
pfam11289	APA3_viroporin	390	moderate
pfam09399	SARS_lipid_bind	387	moderate
pfam11501	Nsp1	371	moderate
pfam12124	Nsp3_PL2pro	371	moderate
pfam12379	DUF3655	370	moderate
pfam12383	SARS_3b	363	moderate
pfam11963	DUF3477	340	moderate
pfam04753	Corona_NS2	322	moderate
pfam01831	Peptidase_C16	315	moderate
pfam05213	Corona_NS2A	276	moderate
pfam13563	2_5_RNA_ligase2	245	moderate
pfam10469	AKAP7_NLS	238	moderate
pfam16688	CNV-Replicase_N	224	moderate
pfam02398	Corona_7	176	moderate
pfam10943	DUF2632	92	moderate
pfam12093	Corona_NS8	78	moderate
pfam17072	Spike_torovirin	70	moderate
pfam03905	Corona_NS4	51	moderate
pfam05528	Coronavirus_5	48	moderate
pfam07204	Orthoreo_P10	27	rare
pfam00035	dsrm	16	rare
pfam04694	Corona_3	14	rare
pfam11030	Nucleocapsid-N	12	rare
pfam00943	Alpha_E2_glycop	7	rare
pfam00270	DEAD	7	rare
pfam03622	IBV_3B	7	rare
pfam13238	AAA_18	5	rare
pfam12226	Astro_capsid_p	5	rare
pfam11395	DUF2873	5	rare
pfam00523	Fusion_gly	5	rare
pfam00485	PRK	5	rare
pfam07690	MFS_1	4	rare
pfam04582	Reo_sigmaC	4	rare
pfam01481	Arteri_nucleo	2	rare
pfam06336	Corona_5a	2	rare
pfam00517	GP41	2	rare
pfam08291	Peptidase_M15_3	2	rare
pfam00069	Pkinase	2	rare
pfam07714	Pkinase_Tyr	2	rare
pfam01815	Rop	2	rare
pfam00083	Sugar_tr	2	rare
pfam00704	Glyco_hydro_18	1	rare
pfam01358	PARP_regulatory	1	rare
pfam02123	RdRP_4	1	rare
pfam00429	TLV_coat	1	rare

^a^Pfam domains from Pfam 31.0 at http://Pfam.xfam.org/

^b^The frequency of the domain occurrence in a set of all *Coronaviridae* genome sequences (2,255 entries) retrieved from GenBank on 3 January 2018.

^c^CATD, *Coronaviridae* Absolute Triage Domain; CTD, *Coronaviridae* Triage Domain. See text for details. Abundant, moderate, and rare indicate the frequency of the domain in all *Coronaviridae* genome sequences.

A Random Forest (RF) classification using the Scikit-learn (Pedregosa et al. 2011) RandomForestClassifier module was performed on the initial triage contigs using a full genome *Coronaviridae* genera training set as follows. For all full genomes in each of the six *Coronaviridae* genera, all six open reading frames were translated and peptides ≥ 100 amino acids in length were collected. HMMER3 hmmsearch (Eddy 2011) was applied to the set of peptides, screening against the *Coronaviridae* Pfam domains ever found in the *Coronaviridae* (see Table 2). For domain hits with e-values < 0.01, the Pfam domain scores were collected into an array organized by genera. An RF model using these six genera sets was then trained and built from 1,000 random trees.

Classification of novel query sequences was performed as follows. For each query nucleotide sequence, all six open reading frames were translated and peptides ≥ 100 amino acids in length were collected. HMMER3 hmmsearch (Eddy 2011) was applied to the set of peptides, screening against the database of *Coronaviridae* Pfam domains and for domain hits with e-values < 0.01, the domain scores were collected into an array. An initial triage was performed to retain only contigs with at least one of the five *Coronaviridae* Triage Domains (CTDs, see below) to yield the initial triage contigs. The RF model was then applied on each query contig and the genus level probability was calculated as the mean predicted probabilities of 1,000 random trees in the forest. A CSV table with the probabilities and a heatmap of the same data are the output from the process.

To facilitate the use of this tool for virus discovery and classification, the process of HMMER3 Pfam domain identification, the encoding of the domain content into a matrix and the RF classification against all available *Coronaviridae* genomes was incorporated into a single, platform-independent Docker image (available for download at https://hub.docker.com/r/matthewcotten/cotten_myphan_coronavirus_classification_tool/). The tool can be downloaded and installed on any computing platform (Unix, Mac, Windows) and includes all required dependencies. Further details on installation and running the tool can be found at the Docker hub link.

### 2.6 Forty-one virus mock contig set

For testing the specificity and sensitivity of the classification methods, a test set of random genome fragments from forty-one virus families including two of each of the six *Coronaviridae* genera was prepared. The set was derived from 492 full genomes (12 genomes from each of 40 virus families) plus 2 genomes from each of the 6 *Coronaviridae* genera. For each genome, 300 random fragments in the size range from 500 nt to full genome size were prepared, combined resulting in a total of 111,577 fragments including 3,316 *Coronaviridae* fragments.

## 3. Results

**Figure 2.**
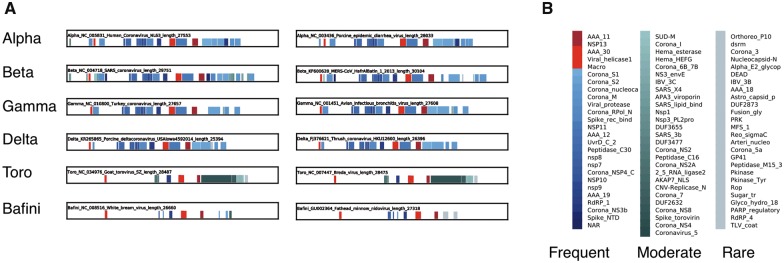
The distribution of Pfam domains across *Coronaviridae* genera. Panel A: Two examples of *Alpha-, Beta-, Gamma-, Delta-, Toro-* and *Bafinivirus* were selected and all protein domains encoded by the full genomes, detected by profile HMMs, were identified and their positions in each virus genome is marked by colored rectangles. Panel B: The *Coronaviridae* Absolute Triage Domains (CATDs) are marked with an red, the *Coronaviridae* Triage Domains (CTDs) are marked with orange, the frequent Pfam domains are marked in shades of blue, the moderately frequent Pfam domains are marked in shades of green and the rare Pfam domains are marked in gray.

The Pfam domain content of all 2,255 available *Coronaviridae* genomes was determined and a variety of distribution patterns were observed, from domains whose frequency across all *Coronaviridae* was >95 per cent, to rare domains present in only a few known genomes. To illustrate this variety, two genomes examples of *Alphacoronavirus*, *Betacoronavirus*, *Gammacoronavirus*, *Deltacoronavirus*, *Torovirus,* and *Bafinivirus* were selected and all domains encoded by the full genomes were identified and their positions in each virus genome is marked by colored rectangles indicating frequent, moderate, or rare occurrence (Fig. 2). The domain content is both extensive and varied by genus and this information might be used to identity and classify *Coronaviridae* sequences.

**Figure 3.**
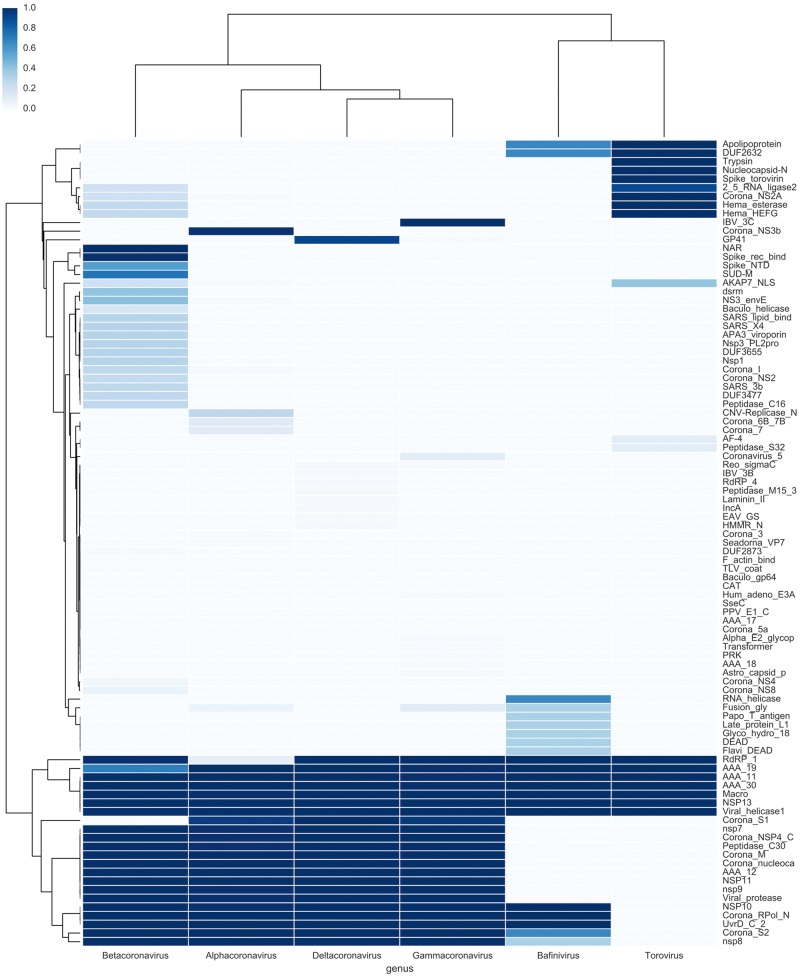
Cluster map of Pfam protein domains encoded by *Coronaviridae* genomes. The protein domain repertoire, as detected by profile HMMs, is plotted as the frequency of each domain in all available full genomes from all *Coronaviridae* genera. Each row represents a protein domain, each column represents a *Coronaviridae* genus. Colors indicate domain frequency within that genus (darkest blue = 1 = all genomes in this genus encode this domain; white = 0 = no genomes in this genus encode this domain, see color bar at upper left).

A hierarchical-clustering of domain content of all genomes in each genus showed three distribution patterns (Fig. 3). Domains present in a high frequency in single genus (upper-third of the cluster map), domains present at high frequency in most or all genera (bottom-third of the cluster map), and domains with low frequency in some genomes or genera (middle of the cluster map). In particular, five domains (AAA_30, Macro, Viral_helicase1, AAA_11, NSP13) were found to be encoded by genomes from all six *Coronaviridae* genera in >95 per cent of the 2,255 *Coronaviridae* genomes examined. We define these domains as *Coronaviridae* Triage Domains (CTDs). Of the five CTDs, three were promiscuous (encoded in all *Coronaviridae* genomes as well as in other virus families), while two domains appeared specific for *Coronaviridae* (AAA_11 and NSP13, termed *Coronaviridae* Absolute Triage Domains (CATD)) and were encoded in all *Coronaviridae* genomes, but were not found in genomes from forty other common virus families infecting animals (results not shown).

**Figure 4.**
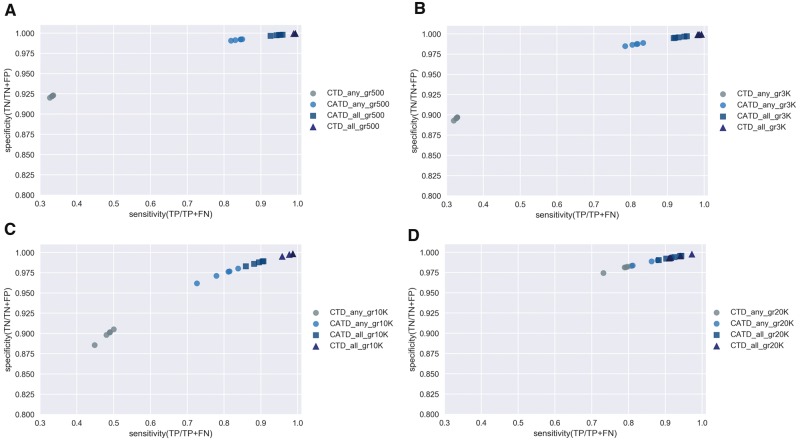
Sensitivity and specificity plot of various triage conditions. The HMM domain content of the forty-one virus mock contig set (111,577 viral genome fragments including 3,316 *Coronaviridae* fragments) was determined for each fragment. The CTD or CATD domain content plus the contig length (≥500 nt, ≥3,000 nt, ≥10,000 nt, ≥20,000 nt) were used as a triage to classify fragments as ‘*Coronaviridae*’ or ‘not *Coronaviridae*’. The contigs classified as *Coronaviridae* for each triage condition were then identified to the genus level using RF classification. The sensitivity (true positive/true positive + false negative) and specificity (true negative/true negative + false positive) for each combined triage and classification method were determined based on the original identity of the input genomes. Panel A. RF classification after triage by 500 nt or larger and CTD or CATD content. Panel B. As in A but with 3,000 nt or larger contigs. Panel C. As in A but with 10,000 nt or larger contigs. Panel D. As in A but with 20,000 nt or larger contigs. Each colored node represents the outcome of one complete triage/classification cycle, each combined method was repeated five times.

The utility of domain content for *Coronaviridae* classification was first tested by developing a simple triage method to identify potential *Coronaviridae* sequence contigs. Preliminary work identified four triage conditions as useful for this purpose. These triage conditions were CTD_any (the contig encodes at least one of the five CTDs), CTD_all (the contig encodes all five CTDs), CATD_any (the contig encodes at least one of the two CATDs) CATD_all (the contig encodes both CATDs). The presence of these domains was combined with contig length cutoffs of 500 nt, 3,000 nt, 10,000 nt, and 20,000 nt.

The performance of these triage conditions for identifying coronavirus contigs was examined. Using a mock contig set derived from forty-one virus families (see Section 2), the Pfam domain content of each fragment was determined using HMMER3 and used to sort the fragments into *Coronaviridae* groups based on fragment length plus CTD and CATD domain content. The accuracy of the classification was assessed in comparison to the classification of the original genome annotation in GenBank (Fig. 4). We ran each classification process five times to control for the random selection of features.

The analyses were run with four size cutoff classes and sensitivity/specificity values for correct classification were color and shape coded for Triage method (≥500 nt panel A, ≥3,000 nt panel B, ≥10,000 nt panel C, and ≥20,000 nt panel D, Fig. 4). The highest sensitivity/specificity values were obtained with the CTD_all triage for all four size classes (dark blue triangles, Fig. 4). Overall performance was observed in the order CTD_any < CATD_any < CATD_all < CTD_all. The combination of triage with either the CTD_all or CATD_all triage, with a 20,000-nt size cutoff and RF classification using all *Coronaviridae* domains resulted in classification of *Coronaviridae* sequence fragments with both sensitivity > 0.9 and specificity > 0.975.

**Figure 5.**
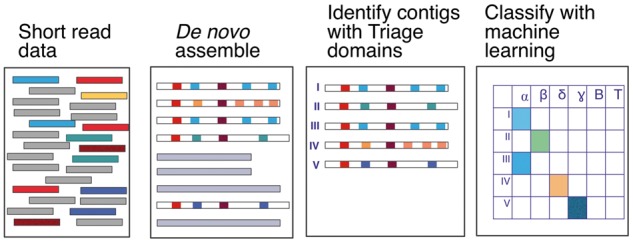
Workflow of the *Coronaviridae* classification tool to identify *Coronaviridae* genomes in NGS data. First, short read NGS data from surveillance samples were *de novo* assembled into larger contigs using SPAdes. Subsequently, putative *Coronaviridae* genome sequences were identified by their encoded triage domains (contig length > 10,000 nt and the presence of at least one CTD) followed by machine learning classification (using RF) to the *Coronaviridae* genus level.

**Figure 6.**
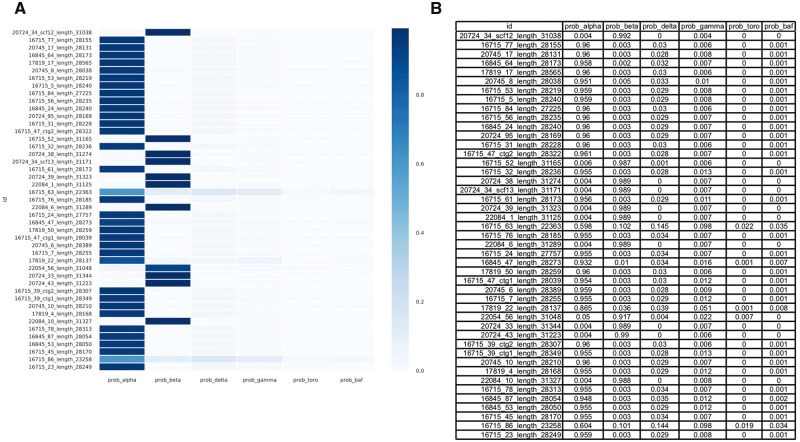
Identification of *Coronaviridae* genomes. *De novo* assembled contigs from rat and bat sample data sets were processed using a triage (contig length > 10,000 nt and the presence of at least one CTD) followed by RF classification to the *Coronaviridae* genus level. About forty-five samples contained *Alphacoronavirus* and *Betacoronavirus* sequences with probabilities > 0.5 (darker blue in the heatmap). These sequences were included in the complete set of samples processed for full genome coronavirus handling. Panel A. Heatmap of predicted *Coronaviridae* genus probabilities. Panel B. Table of probabilities prediction.

We next applied this protein domain-based method to classify *Coronaviridae* genomic sequences generated from next-generation sequencing surveillance data. The NGS data were derived from bat and rat fecal samples collected in the Dong Thap province (Fig. 1) and processed for agnostic, random primed NGS as described in Section 2. All *de novo* assembled *Coronaviridae* contigs that passed the quality control and minimum length cutoff were subjected to Pfam domain content identification, triage by CATD content and length and RF classification. This process is summarized in Fig. 5. The process identified thirty-four potential *Coronaviridae* genomes from 177 bat fecal samples and 11 *Coronaviridae* genomes from 391 rat fecal samples. These forty-five genomes were classified to the *Coronaviridae* genus level using the *Coronaviridae* classification tool (Fig. 6).

Note that screening using specific PCR targeting the conserved RNA-dependent RNA polymerase (RdRp) gene had previously identified CoV sequences in some of the same samples (29). The RdRp sequences generated in that study were closest to the bat *Alphacoronavirus* NC_009657 (*Scotophilus* bat coronavirus 512) and the rat *Betacoronaviruses* NC_026011 (*Betacoronavirus* HKU24) or KF294372 (Longquan Rl rat coronavirus). These are likely to be the same viruses described here at the full genome level.

**Figure 7.**
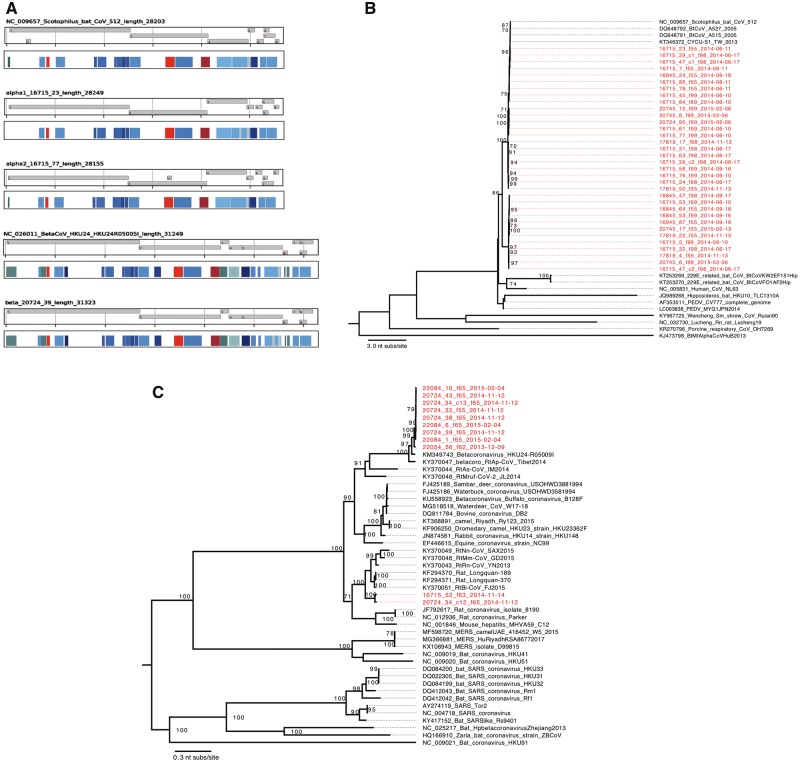
Analyses of identified coronavirus genomes. Panel A. Open reading frames and domain content of the three classes of coronavirus identified in this study. All open reading frames > 130 amino acids in length and the Pfam domains are displayed for an example reported genome from each of the lineage 1 and 2 of *Alphacoronavirus*, and *Betacoronavirus* plus the closest known genomes (*Alphacoronavirus Scotophilus* bat CoV 512, NC_009657 and *Betacoronavirus* strain HKU24, NC_026011). Panel B. Maximum-likelihood phylogenetic tree of the spike protein coding sequences from *Alphacoronaviruses* from this study (highlighted in red) plus selected reference sequences. The tree is mid-point rooted for clarity and only bootstraps ≥70 per cent are shown. Horizontal branch lengths are drawn to the scale of nucleotide substitutions per site. Panel C. Maximum-likelihood phylogenetic tree of the spike protein coding sequences from *Betacoronaviruses* plus a collection of spike coding regions from relevant *Betacoronaviruses.* The tree is mid-point rooted for clarity and only bootstraps ≥70 per cent are shown. Horizontal branch lengths are drawn to the scale of nucleotide substitutions per site.

Two lineages of *Alphacoronavirus* were identified in the surveyed bat samples, one group showed some relationship to the *Scotophilus* bat coronavirus 512 (GenBank NC_009657, Tang et al. 2006) with 96 per cent shared identity across the genome. The other group of *Alphacoronavirus* was distant from any known *Alphacoronavirus* strains and may represent new species. Two groups of *Betacoronavirus* were identified in rat samples, the closest available virus genome in GenBank was the *Betacoronavirus*_HKU24 (KM349743) and the Longquan mouse coronavirus (KF294357) with 95 and 94 per cent shared identity across the entire genome. The next closest coronaviruses were the human coronavirus OC43 and the porcine hemagglutinating encephalomyelitis virus (PHEV) with ∼70 per cent shared amino acid identity across the entire genome. The genome organization for the new coronaviruses was similar to the closest reference genomes sharing similar open reading frame organization as well as similar Pfam domains (Fig. 7A). Furthermore, the expected ribosome slippage sequences between the ORF 1 A and 1AB, as well as the repeat sequences and protease cleavage sites were all present in the new CoVs genomes (results not shown).

To examine the relationship between the reported viruses and known *Alpha*- and *Betacoronaviruses*, the spike protein encoding regions of these genomes were compared with the spike coding regions from the most closely related coronavirus genomes from GenBank. Consistent with observation at the full genome scale, phylogenetic analyses suggested that the Vietnamese bat *Alphacoronaviruses* belonged to two lineages; viruses in the one lineage are closely related (sharing 94–96 per cent nt identities) to *Scotophilus* bat CoVs strains A515 (DQ648719), A527 (DQ648791), CYCU-S1/TW/2013 (KT346372), and 512 (NC_009675), while viruses in the second lineage were more distantly related (sharing 75–76 per cent nt identities) to the four previously mentioned bat CoVs strains (Fig. 7B). The CoVs identified from Vietnamese rats were classified as *Betacoronavirus* and belonged to two distinct lineages as shown in phylogenetic tree (Fig. 7B); viruses from one lineage closely related to the CoV strain HKU24 from Hongkong (KM349743, Lau et al. 2015), while viruses in the second lineage are more related to the rat CoV strains Longquan-189 (KF294370) and -370 (KF294371) (Wang et al. 2015) and rat CoVs from China (KY370051, KY370049, KY37048, and KY370043).

## 4. Discussion

Members of the *Coronaviridae* family of viruses cause health problems in a variety of animal hosts. MERS-CoV often moves from camels to humans (Memish et al. 2014; Dudas et al. 2018) and can spread in health care systems with serious consequences (Assiri et al. 2013). SARS-CoV moved from civet cats to humans and caused substantial morbidity and mortality before it was brought under control (Poon et al. 2004). Several porcine coronaviruses cause frequent problems including PEDV (Kocherhans et al. 2001) and swine acute diarrhea syndrome coronavirus (SADS-CoV) (Zhou et al. 2018). Given the frequent association of *Coronaviridae* members with severe diseases, a more comprehensive description of *Coronaviridae* diversity, especially in animals with frequent human contact, is an important objective.

We describe a *Coronaviridae* sequence classification strategy based on the set of protein domains encoded by the genome sequence. The classification is not dependent on a single domain, but rather the composite score of all domains present in the query sequence. This is a strength of the method that can limit false positive identifications which might be due for example, to shorter regions of homology to a bacterial or host or repetitive sequence. The requirement for longer sequence contigs is also a weakness of the method as sufficient query sequence must be available to encode multiple protein domains. This also limits the tool to assembled contigs rather than short read data. In other words, the sensitivity of the classification is directly dependent upon the length of the genomic sequences, that is, higher sensitivity of genus assignment with longer or complete genome sequence. The classification tool provides a robust, rapid, and alignment-free method to classify large sets of more distantly related sequences. Once a database is generated, the algorithm can be used in the field or resource-limited settings and the classification can be performed with typical contig sets within minutes on a standard laptop. With the availability of the platform independent Docker version of the algorithm (see Section 2), scientists can easily run the analyses on any computing platform.

Given the large number of genomes available for most of the *Coronaviridae* genera, this domain-based classification method can provide a sensitive measure of genome and annotation quality. One consideration is that the genus classification may be broad and the diversity within that genus includes genomes with more distant variations in the protein domains. An additional consideration is that the genus classification of individual *Coronaviridae* genomes in GenBank may not be correct (mis-annotation), that the genome sequences may include errors (machine errors, PCR errors, chimeric sequences) or have been assembled incorrectly or with sequence duplications or deletions (mis-assemblies). The domain method described here can help identify these patterns.

Bats have been suggested to harbor great diversity of CoVs and play a key role in the emergence and transmission of pathogenic CoVs causing severe diseases in human (Menachery, Graham, and Baric 2017). Rats, on the other hand, represent the largest order of mammalian species and are potentially a major zoonotic source of human infectious diseases (Meerburg, Singleton, and Kijlstra 2009; Luis et al. 2013). As part of a large-scale zoonotic surveillance in Vietnam (Rabaa et al. 2015), we applied agnostic deep sequencing to 177 bat and 391 rat samples from a single location. The sample collection was from the Dong Thap province in southern Vietnam where humans and domestic and farm animals live in close proximity. The site is 154 km from Ho Chi Minh City, the largest city in south of Vietnam. From this modest sample size surveillance, forty-four complete or nearly complete genomes belonging to *Coronaviridae* family were identified, thirty-four of which were from bat samples belonging to the *Alphacorovirus* genus and eleven genomes from ten rat samples belonging to the *Betacoronavirus* genus. The bat fecal samples were pooled material from five to ten individuals, thus the total individual bats samples screened ranged from 885 to 1,770 and the frequency of full genome identification was 1.9–4.0 per cent (34/1,770 to 34/855). In comparison, the 391 screened rat samples were derived from individual fecal pellets and frequency of the coronavirus genome identification was 2.8 per cent (11/391). Given this small sample size, the frequency of CoVs identified was not strikingly different between bats and rats.

While it is possible to use Blast methods to identify/classify the putative viral genomes, one advantage of the tool described here is that it rapidly provides a genus-level classification, which is not directly obtained from a Blast search. Certainly, one could use a Blast search to find the closest sequence in a database and then use this as a classification if (1) the closest homology sequence covers 100 per cent of the length of the query sequence, and (2) the closest entry contains sufficient classification annotation. Finally, we are not presenting this new tool as a replacement for Blast (which is a reliable and highly trusted tool). Instead, the domain-based classification method described here provides a rapid, alternative and complementary classification method that can help organize complex data sets.

Of note, this is the first whole genome characterization of *Alpha*- and *Betacoronaviruses* from rats and bats from southern Vietnam. Although there are reports in the literature of enhanced CoV prevalence in various bat species (Anthony et al. 2017), at least in the current survey, when the same agnostic sequencing approach was applied to both bat and rat samples, similar virus frequencies were observed, suggesting that rats and in more general rodents may be as important a host for CoVs as bat species. Indeed surveys using similar agnostic NGS methods have identified a wide range of virus diversity in rats and other rodents (Li et al. 2015, 2016; Wang et al. 2015, 2017). The zoonotic potential of these reported viruses is yet to be defined. Prior to the current study, only single examples of these viruses were available and the availability of a larger number of full genomes for these viruses will facilitate more functional studies.

Sensitive surveillance that is not dependent on known viral sequences for primer design is important for documenting circulating viruses and for identifying those that might cause problems in the future. This is particularly of great importance for virus discovery and to characterize potentially zoonotic viruses in exotic hosts that have not been described. The bat *Alphacoronaviruses* described here had only a single genome available in GenBank and we have enriched the public database with thirty-four additional genomes. The rat *Betacoronaviruses* also provide description of novel, more distant members of a recognized but poorly characterized group of CoVs. The disease potential of these viruses in their normal host and their zoonotic potential are yet to determine. Certainly, the reported sequences would add important information to the growing collection of coronavirus diversity and particular with great relevance in the context of south and east Asia where SARS-CoV first emerged. We hope they are useful for future studies, for monitoring disease of unknown origin and for increasing our understanding of the molecular diversity of these viruses.

## Accession numbers

The coronavirus sequences described in this study have been deposited in GenBank under the accession numbers MH687934–MH687978.
